# Incidence and predictive factors of hyperventilation syndrome in patients after COVID 19 pneumonia: a prospective cohort study

**DOI:** 10.12688/f1000research.152196.1

**Published:** 2024-12-06

**Authors:** Hela CHERIF, Salma Mokaddem, Soumaya Debiche, Slim Kalboussi, Ferdaous Yangui, Mohamed Ridha Charfi

**Affiliations:** 1 Pulmonology Department, Internal Security Forces Hospital, Tunis, Tunisia; 2 Faculty of Medicine of Tunis, University of Tunis El Manar, Tunis, Tunis, Tunisia; 3 Research Laboratory on Health and Environment for security Foces LR21INT01, Tunis, Tunisia

**Keywords:** Hyperventilation Syndrome; COVID-19 pneumonia; Post-Traumatic Stress Disorder; Prospective Studies; Incidence

## Abstract

**Background:**

This study investigates the incidence and predictive factors of Hyperventilation Syndrome (HVS) in patients after COVID 19 pneumonia, addressing the clinical overlap between these conditions.

**Methods:**

A one-month prospective study was conducted, tracking survivors of COVID-19 pneumonia. Patients were evaluated for ongoing clinical status, including HVS and post-traumatic syndrome disorder (PTSD), using clinical questionnaires, mMRC, Post-COVID-19 Functional Status (PCFS) Score, Nijmegen score, and PTSD Checklist for DSM-5 questionnaire.

**Results:**

Our study included 222 patients (median age: 57 years, male predominance 62.6%). Somatic comorbidities, primarily metabolic disorders, were reported in 71.2% of cases. The majority had severe or critical infection forms (78.4%), and 91.9% experienced acute symptoms, with 86.5% having three or more symptom clusters. At one month follow-up, dyspnea (52.9%) and asthenia (21.7%) persisted. Functional limitations (PCFS Grade > 2) were observed in 19.6% of patients. The overall incidence of HVS was 158 per 1000 patients, and PTSD was 445 per 1000 patients. Multivariate logistic regression identified cognitive impairment (acute phase), persistent weight loss (post-COVID-19 phase), PCFS grade > 2, and PTSD as independent factors for developing HVS, with relative risks (RRs) of 3.47 (95%CI [1.48-8.31]; p = 0.004), 11.87 (95%CI [1.25-112.88]; p = 0.031), 3.24 (95%CI [1.34-7.86]; p = 0.009), and 5.98 (95%CI [2.27-15.77]; p < 0.001), respectively.

**Conclusion:**

HVS is prevalent in the post-COVID-19 phase, affecting 15.6 % of survivors. Identified predictive factors suggest the convergence of psychosomatic pathophysiological mechanisms. Further research is crucial for a detailed understanding of these mechanisms in long COVID-19 patients.

**Table:** 

**What is known on this topic**
▪ COVID-19 sequels are heterogeneous encompassing multiple incapacitating symptoms that overlap with signs of inappropriate hyperventilation. ▪ Few case series have reported the diagnosis of hyperventilation syndrome in survivors of post-acute COVID-19 pneumonia.
**What this study adds**
▪ This is the first prospective cohort study assessing the exact incidence of hyperventilation syndrome at 156 per 1000 patients experiencing post COVID-19 residual symptoms. ▪ The predictive model for developing hyperventilation syndrome post-COVID-19 includes cognitive impairment in the acute phase, persistent weight loss in the post-infection phase, functional alteration (PCFS grade > 2), and PTSD. ▪ The predictive factors for hyperventilation syndrome highlight the convergence of psychosomatic pathophysiological mechanisms.
**Research perspectives**
▪ Further investigations, particularly in neurophysiology and psychology, are imperative to elucidate the precise mechanisms promoting hyperventilation syndrome after COVID-19 pneumonia. ▪ The development of individualized management strategies is warranted to enhance the care and well-being of individuals grappling with the lasting effects of COVID-19.

## Introduction

Covid-19 acute pneumonia presents a diverse clinical spectrum, ranging from asymptomatic cases to severe and critical forms. ^1, 2^ Characterized by acute respiratory symptoms, it may also exhibit nonspecific multisystemic manifestations such as fever, gastrointestinal issues, and neuropsychiatric signs, forming distinct symptom clusters. ^1, 3, 4^ While the acute phase is extensively documented, attention has shifted to the significant concern of the consequences of COVID-19. ^5, 6^

Hyperventilation Syndrome (HVS) is delineated by an array of somatic symptoms induced by physiologically inappropriate and reproducible hyperventilation, either entirely or partially instigated by voluntary hyperventilation. ^7– 10^ The estimated prevalence of HVS ranges from 6% to 10% within the general population, predominantly afflicting women. ^7, 11^ Operating as an exclusion diagnosis, HVS may manifest as an acute crisis triggered by physical or psychological stress, mimicking medical emergencies such as acute coronary syndrome or hypocalcemic spasms. This acute presentation is marked by agitation, dyspnea, and symmetrical paresthesias. In its chronic forms, although more prevalent, diagnosis proves challenging as the hyperventilation component may not be prominently featured. Patients may manifest general indicators such as chronic fatigue, headaches, dyspnea, cough, atypical chest pain, palpitations, or peripheral vasospasm. ^7, 10, 12– 14^

Given the significant clinical symptom overlap between long COVID-19 and HVS, some authors suggest that HVS may play a notable role in the post-COVID-19 phase. ^15, 16^ Therefore, our study aims to determine the incidence rate and predictive factors of HVS within a prospective cohort of survivors of COVID-19 pneumonia.

## Methods

### 1. Study design and settings

This study is a prospective cohort investigation conducted at the Pulmonology Outpatient Department of the Internal Security Forces Hospital, La Marsa, Tunisia, from September 2020 to December 2021. The study focuses on patients admitted for SARS-CoV-2 acute pneumonia, with follow-up examinations conducted one month after the acute COVID-19 infection.

### 2. Participants selection

Eligible participants for this study were individuals aged 18 years and older with a confirmed diagnosis of SARS-CoV-2 pneumonia, who actively participated in post-COVID-19 follow-up appointments. The confirmation of SARS-CoV-2 pneumonia involved the detection of the viral genome in the upper airways using RT-PCR on nasopharyngeal swabs, complemented by a chest CT scan indicating the presence of lung damage. Individuals who declined to participate in this study were excluded from consideration.

Exclusions encompassed patients without a confirmed SARS-CoV-2 infection, and those who presented a normal chest CT scan during the acute infection phase. Furthermore, during the follow-up period, patients who did not complete the Nijmegen Questionnaire were excluded from the analysis.

### 3. Data collection and outcomes measures

**3.1. Step 1: Inclusion at hospital discharge**

Clinical data of the enrolled patients were systematically noted. Demographic and clinical features, encompassing age, gender, BMI, cigarette smoking, somatic and psychiatric comorbidities were meticulously obtained. A comprehensive examination of acute COVID-19 pneumonia infection was conducted, focusing on COVID 19-related symptoms. Patients were asked about these symptoms, which were categorized into five symptomatic clusters:

▪ Constitutional Signs: Fatigue, fever, and weight loss
▪ Cardiorespiratory Cluster: Dyspnea, cough, palpitations, and chest pain
▪ Gastrointestinal Cluster: Nausea, vomiting, and diarrhea
▪ Musculoskeletal Cluster: Myalgia and arthralgia
▪ Neuropsychiatric Cluster: Dysgeusia, anosmia, headache, dizziness, and cognitive impairment

Additionally, dyspnea was assessed using the modified Medical Research Council (mMRC) dyspnea scale. Markers of the severity of acute COVID-19 pneumonia were noted, including therapeutic features such as the intensity of oxygen requirement (low or high-flow oxygen therapy), admission to the intensive care unit (ICU), anticoagulation therapy, and the occurrence of complications, particularly pulmonary embolism.

**3.2. Step 2: One-month follow-up appointment**

Patients’ one-month follow-up appointments were scheduled following their discharge date and communicated to them via phone calls. Key outcome measures assessed during this examination included:

**Evaluation of the ongoing clinical status after recovery from acute COVID-19:**

▪ Post-COVID-19 Follow-up Questionnaire: Patients were queried about symptoms related to SARS-CoV-2 infection, assessing both residual and newly developed symptoms. Response options were recorded as “yes” or “no”. This questionnaire maintained the same five symptomatic clusters as in the initial assessment.
▪ mMRC Dyspnea Scores: Dyspnea evaluation was tracked using the mMRC dyspnea scale to monitor changes over time.
▪ Post-COVID-19 Functional Status (PCFS) Score ^17^: All patients were administered the validated Arabic version of the PCFS questionnaire, categorizing individuals into five grades based on the degree of functional limitations experienced.

**Screening for Hyperventilation Syndrome:** The Nijmegen score, assessing the clinical probability of HVS, was administered to all patients during the one-month follow-up. A score higher than 23/64 indicated the presence of HVS. ^12, 18^

**Screening for Post-Traumatic Stress Disorder (PTSD):** COVID-19 survivors were screened for PTSD using the PTSD Checklist for DSM-5 (PCL-5) questionnaire, which has been validated in Arabic. A PCL-5 cutoff score higher than 33/80 suggested probable PTSD. ^19^

**Arterial Blood Gas Test (ABG):** Patients underwent an ABG test to measure post-recovery blood levels of various gases, including oxygen (O2) and carbon dioxide (CO2).

### 4. Data analysis

The collected data underwent thorough statistical analysis, with due consideration for variable normality assessed using the Kolmogorov-Smirnov normality test. Nominal values were presented as frequencies and percentages, while continuous non-Gaussian variables were expressed using medians (M) and quartiles [Q1-Q3].

To discern predictive factors for the occurrence of HVS, the sample was divided into two groups: patients with HVS (HVS+) and patients without HVS (HVS-). Initial analytical comparisons were conducted between these two groups to reveal factors associated with HVS. Continuous variables were compared using the Mann-Whitney U test, whereas discrete variables were assessed using the chi-squared or Fisher’s exact test, as appropriate.

Subsequently, a multivariate logistic regression model was employed to evaluate the influence of explanatory variables on the incidence of HVS. Coefficients (β) associated with each independent variable were estimated, and relative risks (RRs) were calculated by exponentiating these coefficients (exp(β)). A 95% confidence interval (95% CI) for each relative ratio was computed to gauge the precision of the estimate. The overall fit of the model was evaluated using the Hosmer-Lemeshow test, with a non-significant result (p > 0.05) indicative of an acceptable fit. Additionally, the model’s explanatory power was assessed through the calculation of the Adjusted R ^2^.

For each value, the data was tabulated using the Microsoft Excel software. All statistical analyses were conducted with a significance threshold set at a p-value of 0.05 considered significant for all data analyses.

### 5. Ethical considerations

This study involving human subjects is ethically sound. Participants were thoroughly briefed on procedures and objectives, providing verbal consent. It was elected to minimize physical contact and the handling of documents, thereby reducing the risk of transmission in the context of the ongoing epidemic at the time of the study Data confidentiality is assured, adhering to rigorous ethical standards. Ethical committee approval was obtained from the Ethical committee of the FSI hospital under the approval number 05/2020 on September16, 2020.

## Results

### 1. Baseline patient characteristics

Our prospective cohort comprised 222 patients hospitalized for SARS-CoV-2 acute pneumonia. Table 1 provides a summary of their baseline demographic and clinical characteristics. The median age was 57 years [50-67], with a male predominance of 62.6%. Among participants, 35.6% were ever smokers. The average BMI was 29.7 kg/m ^2^ [26.8-32.8]. Notably, 48.1% of patients were classified as obese, and 39.6% were overweight.

**Table 1.** Baseline Demographic and Clinical Features in the Studied Cohort.

		All cohort (n=222)	Patients with HVS (n=35)	Patients without HVS (n=187)	p-value
Gender	**Male**	62.6%	48.6%	65.2%	0.061
	**Female**	38.3%	51.4%	34.8%	
Age (years)		57 [50-67]	58 [51-67]	57 [49-67]	0.827
BMI (Kg/m ^2^)		29.7 [26.8-32.8]	31.2 [28.0-33.1]	29.4 [26.7-32.8]	0.160
Vaccinated		24.8%	25.7%	24.6%	0.888
EverSmoker		35.6%	30.3%	39.9%	0.300
Comorbidities					
OverallComorbidities	**None**	28.8%	14.3%	31.6%	0.038*
	**≥1**	71.2%	85.7%	68.4%	
Anxiety		5.4%	11.4%	4.3%	0.086
Diabetes		35.1%	25.7%	36.9%	0.203
Hypertension		41.9%	51.4%	40.1%	0.213
Obesity		48.1%	63.6%	45.3%	0.052
CoronaryHeartDisease		7.2%	11.4%	6.4%	0.293
Dyslipidemia		19.4%	28.6%	17.6%	0.133
Asthma		8.6%	17.1%	7%	0.048*

HVS: Hyperventilation syndrome; BMI: Body Mass Index; Continuous variables are expressed in median and quartiles M [Q1-Q3]; Bold p values indicate significant results (p<0.05);

Somatic comorbidities were reported in 71.2% of cases, predominantly metabolic disorders. Following obesity, the most prevalent comorbidities were hypertension (41.9%), diabetes (35.1%), and dyslipidemia (19.4%). Cardiorespiratory diseases were less common, with asthma noted in 8.6% and coronary heart disease in 7.2% of patients. A history of anxiety was reported in 5.6% of patients.

### 2. Acute features of COVID-19 pneumonia

Symptoms manifested in 91.9% of the enrolled patients, with 86.5% experiencing three or more symptom clusters, as detailed in Table 2. Predominantly, constitutional (94.1%), cardiorespiratory (92.8%), and neuropsychiatric (79.7%) signs emerged as the most prevalent clusters during the acute phase. Dyspnea and fatigue ranked highest with incidences of 82.4% and 80.5%, respectively. Individuals reporting dyspnea frequently exhibited severe forms of mMRC 3 and 4 in 24.9% and 45.2% of cases, respectively. Approximately two-thirds of participants also reported a weight loss of around 6 kg [3-10].

**Table 2.** Clinical Presentation in Patients with Acute COVID-19 Pneumonia and during the Post-COVID-19Phase.

COVID symptoms	Acute Phase of COVID-19 Pneumonia				One-month post-COVID-19pneumonia			
	All Cohort (n=222)	Patients without HVS (n=187)	Patients with HVS (n=35)	p-value	All Cohort (n=222)	Patients without HVS (n=187)	Patients with HVS (n=35)	p-value
Constitutionalsigns	94.1%	95.7%	85.7%	0.021*	23%	23,0%	22.9%	0.986
Fatigue	80.5%	80.6%	80%	0.930	21.7%	18.8%	37.1%	0.016*
Fever	66.1%	65.6%	68.6%	0.733	0.5%	0.5%	0%	0.664
Weightloss	67%	66.7%	68.6%	0.826	2.3%	1.1%	8.6%	0.006*
Weightloss (Kg)	6 [3-10]	6 [3-10]	5 [3-10]	0.206	-	-	-	-
Cardiorespiratory cluster	92.8%	92.5%	94.3%	0.710	62.2%	62.6%	60%	0.774
Chest pain	41.2%	38.2%	57.1%	0.036*	19.9%	19.4%	22.9%	0.634
Cough	57%	57.5%	54.3%	0.722	13.1%	11.3%	22.9%	0.063
Palpitations	35.3%	33.9%	42.9%	0.307	13.6%	10.8%	28.6%	**0.005**
Dyspnea	82.4%	81.2%	88.6%	0.293	52.9%	52.7%	54.3%	0.862
Dyspnea stages								
mMRC 1	14.7%	15%	13.3%	0.331	44.5%	47%	31.6%	0.542
mMRC 2	15.3%	16.3%	10%		45.4%	43%	57.9%	
mMRC 3	24.9%	26.5%	16.7%		8.4%	8%	10.5%	
mMRC 4	45.2%	42.2%	60%		1.7%	2%	0%	
Gastrointestinal Cluster	50.5%	52.4%	40%	0.178	5.9%	5.9%	5.7%	0.969
Nausea and vomiting	32.6%	32.3%	34.3%	0.814	2.3%	2.2%	2.9%	0.796
Diarrhea	37.7%	35.7%	48.6%	0.149	0.5%	4.8%	5.7%	0.827
Musculoskeletal cluster	73.4%	74.9%	65.7%	0.261	28.4%	28.9%	25.7%	0.703
Myalgia	67%	66.7%	68.6%	0.826	20.4%	18.3%	31.4%	0.076
Arthralgia	58.8%	57.5%	65.7%	0.367	20.4%	18.3%	31.4%	0.076
Neuropsychiatric Cluster	79.7%	82.4%	65.7%	0.025*	40.5%	40.6%	40%	0.943
Headache	58.4%	57.5%	62.9%	0.557	15.4%	13.4%	25.7%	0.065
Dizziness	40.7%	38.2%	54.3%	0.075	14.9%	11.8%	31.4%	0.003*
Cognitive impairment	41.4%	36.2%	68.6%	<0.001*	25%	20.5%	48.6%	<0.001*
Dysgeusia	40.7%	37.6%	57.1%	0.031*	4.1%	3.8%	5.7%	0.592
Anosmia	38.5%	34.9%	57.1%	0.013*	5.4%	4.3%	11.4%	0.088
Overall Clusters								
Asymptomatic	0.9%	0.5%	2.9%	0.101	23.4%	23%	25.7%	0.194
1 cluster	3.2%	2.1%	8.6%		34.2%	32.6%	42.9%	
2 clusters	9.5%	9.1%	11.4%		18.5%	20.9%	5.7%	
≥ 3 clusters	86.5%	88.2%	77.1%		23.9%	23.5%	25.7%	

HVS: Hyperventilation syndrome; BMI: Body Mass Index; Continuous variables are expressed in median and quartiles M [Q1-Q3]; Bold p values indicate significant results (p<0.05);

The majority of participants presented with severe or critical infection forms (78.4%). Radiological manifestations of COVID-19pneumonia affected over 50% of the pulmonary parenchyma on chest CT scans in 73.8% of instances. High-flow oxygen therapy was required in 40.3%, while 6% of patients necessitated ICU admission for respiratory support. Pulmonary embolism complicated the course of 10.3% of COVID-19 pneumonias. The median hospital stay in our cohort was 10 days [7-15].

### 3. One-month follow-up evaluation

**3.1. Clinical and functional assessment**

The post-COVID-19 phase marked a substantial improvement in all symptoms, demonstrating a significant decrease in symptom occurrence and cluster cumulation compared to the early infection phase. At the one-month appointment, 23.4% of our cohort was asymptomatic, and 34.2% exhibited mono-cluster symptoms. The cardiorespiratory and neuropsychiatric clusters were the most residual, observed in 62.2% and 40.5% of patients, respectively. The most persistent physical complaints were dyspnea (52.9%) and asthenia (21.7%). Notably, dyspnea presentations were less severe, with 89.9% staged as mMRC 1 or mMRC 2. A quarter of participants still reported cognitive impairment at the one-month follow-up. The Post-COVID-19 Functional Status (PCFS) assessment revealed that 47.4% of patients experienced no functional limitations (Grade 0). Negligible (Grade 1) and slight (Grade 2) functional limitations were observed in 16.3% and 16.7% of examined patients, respectively. Severe limitations (Grade 4) affected only 4.2% of cases.

**3.2. Screening for HVS and PTSD**

The median Nijmegen Score was 9 points [3-18], with 35 participants having a score suggestive of HVS, resulting in an overall hospital Incidence of 158 per 1000 patients, which translates to 15.8%. Post-traumatic stress disorder (PTSD) screening using PCL-5 indicated an average score of 30 points [23-46], with 44.5% of our cohort displaying psychological distress consistent with a PTSD diagnosis.

**3.3. Arterial blood gas test**

The ABG test was performed on 100 patients. The average PaO2 and PaCO2 were 89 mmHg [81.5-98] and 36.2 mmHg [35-39], respectively. Hypoxemia and hypocapnea were reported in 21% and 24% of ABG tests, respectively. The median bicarbonate concentration was 25 mmol/L [23.5-26], with 17% of tested patients showing levels lower than the limit of normal. The median pH level was 7.43 [7.42-7.45]. Only 4% of patients featured alkalosis.

**Comparison between Two Groups: HVS+ and HVS-**

The overall patient characteristics of both groups revealed general similarities in demographics and clinical features, as detailed in Table 1. There were no significant differences in age, smoking habits, or BMI between the two groups. Although HVS+ patients showed a trend towards female predominance compared to HVS-, this difference did not reach statistical significance (p=0.061). The presence of one or more comorbidities was significantly associated with HVS+ (85.7% versus 68.4%; p=0.038). However, no significant variations were noted in psychiatric or somatic comorbidities between the two groups, except for asthma, which was statistically associated with HVS+ (p=0.048).

**Figure 1.**
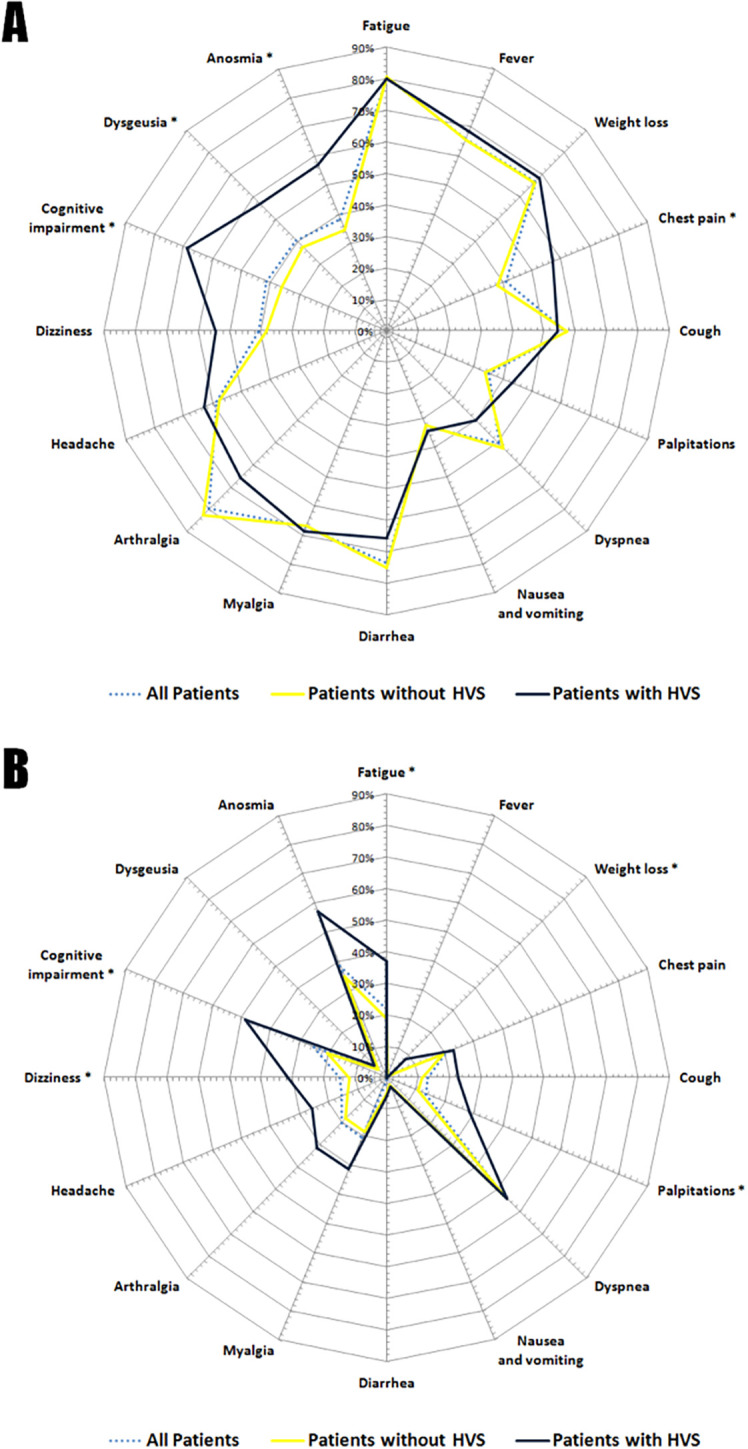
Comparative Radar Chart Illustrating COVID-19 Symptoms in Acute Pneumonia and the Post-Acute Phase. (A): Symptoms during the Acute Phase; (B): Symptoms during the Post-Acute Phase; HVS: Hyperventilation Syndrome. Symptoms marked with an asterisk (*) indicate a significant difference between patients with and without HVS (p<0.05)

In Figure 1 and Table 2, we highlighted the main distinctions between the two groups concerning the clinical presentation of acute COVID-19 pneumonia and symptoms reported one month after. During the early infectious episode, patients with HVS tended to manifest slightly fewer symptoms and less cluster accumulation than patients without HVS. This trend held true for all symptomatic clusters except for the cardiorespiratory cluster (94.3% versus 92.5%; p=0.710), where complaints like dyspnea (p=0.293) and chest pain (p=0.036) were notably more prevalent in this group. Patients without HVS significantly exhibited, in terms of frequency, more symptoms related to neuropsychiatric cluster (p=0.025) during the acute viral infection, even though specific symptoms such as dysgeusia (p=0.031), anosmia (p=0.013), and cognitive impairment (p<0.001) were significantly associated with HVS.

Concerning the post-acute phase, both groups experienced recovery from most symptoms. However, the HVS+ group showed a significantly slower recovery in terms of signs suggestive of HVS, particularly palpitations (p=0.005) and dizziness (p=0.003). Notably, signs unrelated to the classical clinical constellation of hyperventilation, particularly persistent weight loss (p=0.006) and residual cognitive impairment (p<0.001), were observed.

No marker of COVID-19 pneumonia severity was significantly associated with HVS in our cohort ( Table 3). Substantial differences were not observed between both groups in terms of the clinical severity of COVID-19, the radiological extension of COVID-19 lesions, oxygen requirement, ICU admission, the incidence of pulmonary embolism, or hospital stay duration. No significant difference in ABG tests was encountered in both groups either.

**Table 3.** Acute COVID-19 Severity Markers and Post-Infection Sequelae in Our Cohort.

	All Cohort (n=222)	Patients with HVS (n=35)	Patients without HVS (n=187)	p value
Acute COVID-19 PneumoniaMarkers				
Infection Severity				
Mild to Moderate	21.6%	71.4%	79.7%	0.276
Severe to Critical	78.4%	28.6%	20.3%	
Extension of radiologic lesions on initial Chest CT scan				
<10%	3.6%	8.6%	2.7%	0.271
10-25%	22.5%	20%	23%	
25-50%	44.1%	37.1%	45.5%	
50-75%	23.4%	22.9%	23.5%	
>75%	6.3%	11.4%	5.3%	
Oxygenrequirement				
Low O2 flow	59.7%	54.3%	60.8%	0.474
High O2 flow	40.3%	45.7%	39.2%	
ICU Admission	6%	6.5%	5.9%	0.908
PulmonaryEmbolism	10.3%	6.9%	10.9%	0.512
Hospital Stay (days)	10 [7-15]	12.5 [8-15.5]	10 [7-15]	0.084
One-month post-COVID-19 pneumonia				
Post-COVID-19 Functional Status				
Grade 0	47.4%	8.6%	55%	<0.001*
Grade 1	16.3%	22.9%	15%	
Grade 2	16.7%	25.7%	15%	
Grade 3	15.3%	37.1%	11.1%	
Grade 4	4.2%	5.7%	3.9%	
Nijmegen Score (points)	9 [3-18]	33 [25-40]	7 [2-14]	**-**
PCL-5 (points)	30 [23-46]	55 [34-75]	28 [22-41]	<0.001*
PTSD	44.5%	80,00%	37.7%	<0.001*

Arterial Blood Gas	All Cohort (n=100)	Patients with HVS (n=17)	Patients without HVS (n=83)	p-value
PH	7.43 [7.42-7.45]	7.42 [7.40-7.44]	7.43 [7.42-7.45]	0.177
PaO2 (mmHg)	89 [81.5-98]	90 [84-97]	89 [81-98]	0.993
PaCO2 (mmHg)	36.2 [35-39]	39 [35-40.2]	36 [35-39]	0.237
[HCO3-] (mmol/L)	25 [23.5-26]	25 [23.5-26]	24 [23-26]	0.941
SaO2 (%)	97 [96-98]	97 [96-97.4]	97 [96-98]	0.402

Bold p values indicate significant results (p<0.05); HVS: Hyperventilation Syndrome; PTSD: Post-Traumatic Stress Disorder; PCL-5:Posttraumatic Stress Disorder Checklist for DSM-5PaO2: Partial Pressure of Oxygen; PaCO2: Partial Pressure of Carbon Dioxide; [HCO3-]: Bicarbonate Ion Concentration; SaO2: Arterial Oxygen Saturation; ICU: Intensive Care Unit.

Distinctive functional and psychological outcomes were evident between the compared groups at the one-month appointment ( Table 3). Worse PCFS grades were significantly associated with the HVS+ group, as most patients with HVS presented with PCFS grade > 2 (42.8% versus 15%; p<0.001). The PCL-5 score was significantly higher in patients with HVS (55 [34-75] versus 28 [22-41]; p<0.001). A positive diagnosis of PTSD was significantly associated with HVS (p<0.001).

### 4. Predictive factors for HVS

To identify predictive factors for HVS within our cohort, logistic regression analysis was conducted, encompassing various variables, including demographic and clinical factors, acute COVID-19 pneumonia presentation, COVID-19 pneumonia severity markers, persistent symptoms, PCFS, and PTSD. As indicated in Table 4, multivariate analysis revealed that presenting with cognitive impairment during the acute phase, persistent weight loss, PCFS grade > 2, and PTSD were independent factors for developing HVS, with a relative risk (RR) of 3.47 (p = 0.004), 11.87 (p = 0.031), 3.24 (p = 0.009), and 5.98 (p < 0.001), respectively.

**Table 4.** Univariate and Multivariate Logistic Regression Analysis of Factors Influencing theOccurrence of Hyperventilation Syndrome.

	UnivariateLogisticRegression					MultivariateLogisticRegression Model *					
	β	SE	p-value	OR	95% CI	Adjusted β	SE	Wald χ ^2^	p-value	RR	95% CI
Constant	-	-	-	-	-	-3.756	0.527				
Comorbidities ≥ 1	1.017	0.508	0.045	2.77	1.02-7.49						
Acute COVID-19 Phase											
Constitutionalsigns	-1.316	0.603	0.029	0.27	0.08-0.87						
Neuropsychiatric cluster	0.890	0.404	0.028	2.43	1.10-5.38						
Chest Pain	0.770	0.373	0.039	2.16	1.04-4.49						
Cognitive Impairment	1.346	0.395	0.001	3.84	1.77-8.33	**1.244**	**0.433**	**8.23**	**0.004**	**3.47**	**1.48-8.11**
Dysgeusia	0.793	0.374	0.034	2.21	1.06-4.60						
Anosmia	0.909	0.375	0.015	2.48	1.19-5.17						
Post-Phase											
Fatigue	0.936	0.397	0.018	2.55	1.17-5.55						
WeightLoss	2.155	0.933	0.021	8.62	1.39-53.67	**2.475**	**1.149**	**4.64**	**0.031**	**11.88**	**1.25-112.88**
Palpitation	1.200	0.443	0.007	3.32	1.39-7.91						
Dizziness	1.229	0.429	0.004	3.42	1.47-7.92						
Cognitive Impairment	1.296	0.384	<0.001	3.65	1.72-7.76						
PCFS > Grade 2	1.447	0.400	<0.001	4.25	1.94-9.31	**1.175**	**0.452**	**6.75**	**0.009**	**3,24**	**1.34-7.86**
PTSD	1.888	0.449	<0.001	6.61	2.74-15.94	**1.788**	**0.495**	**13.07**	**<0.001**	**5.98**	**2.27-15.77**

* Hosmer-Lemeshow test for model fit (χ ^2^ = 1.379, p = 0.947). Adjusted R ^2^ = 0.32; Significance is determined for all p-values < 0.05; SE: Standard Error; RR: relative risk RR; CI: Confidence Interval; PCFS: Post COVID-19 Functional Status Scale; PTSD: Post-Traumatic Stress Disorder.

The probability (p) of developing HVS based on this multivariate logistic regression model is represented by the following equation:

$$\text{logit}\;\left(p\right)=-3.756+1.244*X_{1}+2.475*X_{2}+1.175*X_{3}+1.788*X_{4}$$

where:

$$X_{1}:\text{Cognitive impairment during the acute COVID}-19\;\text{phase}\;\left(\;1\;\text{if present},0\;\text{if absent}\right)$$

$$X_{2}:\text{Weight loss during the post}-\text{COVID}-19\;\text{phase}\;\left(\;1\;\text{if present},0\;\text{if absent}\right)$$

$$X_{3}:\text{PCFS grading}\;\left(1\;\text{if PCFS}>2,0\;\text{if PCFS}\leq 2\right)$$

$$X_{4}:\text{PTSD during the post}-\text{COVID}\;19\;\text{phase}\;\left(\;1\;\text{if present},0\;\text{if absent}\right)$$

The logistic regression model’s adequacy was assessed through the Hosmer-Lemeshow test, yielding a non-significant result (χ ^2^ = 1.379, p = 0.947), suggesting a satisfactory fit of the model to the data. The model’s explanatory power was further evaluated using the Adjusted R ^2^, revealing a value of 0.32. This indicates that approximately 32% of the variance in the occurrence of HVS in the post-COVID-19 phase is accounted for by these four independent factors.

## Discussion

Despite considerable scientific enthusiasm and extensive medical investigations into the clinical aspects and therapeutics of both acute infection and persistent COVID-19 symptoms, researchers have paid limited attention to HVS in survivors of COVID-19 pneumonia. ^16, 20^ This study sheds light on this topic through a prospective cohort study involving 222 patients and showed that HVS is prevalent in the post-COVID-19 phase, affecting 15.6% of survivors. Identified predictive factors suggest the convergence of psychosomatic pathophysiological mechanisms.

The mechanisms underlying symptoms in HVS remain incompletely understood. Authors posit that hypocapnia and alkalosis induced by inappropriate hyperventilation play a crucial role. Increased ventilation leads to dyspnea due to the muscular effort it engenders, creating a cycle of stress that perpetuates the process. ^7, 21, 22^ Furthermore, symptoms potentially related to hypocapnia are inherently anxiogenic. This scenario is frequently observed in response to various stimuli, both organic and psychiatric, such as exercise or stress, imparting psychosomatic components to this syndrome. ^13, 23^

To the best of our knowledge, this cohort study is the inaugural contribution in the literature assessing the incidence of HVS in a substantial sample of patients after COVID-19 pneumonia. Our estimation places the incidence of 156 per 1000 patients hospitalized for COVID-19 pneumonia., surpassing the classical reported prevalence of 6-10% in the general population. ^7, 11^ Previous studies examining post-COVID-19 patients have been confined to limited case series with cross-sectional designs, thus lacking sufficient statistical power to accurately ascertain the incidence of this condition. ^15, 16, 20, 24^ While HVS is traditionally associated with a higher prevalence in females, with a male-to-female sex ratio ranging from 1:2 to 1:4, our findings suggest an equal gender distribution in survivors of COVID-19. ^7, 11^

Within our cohort, patients with somatic comorbidities exhibit a significant susceptibility to developing HVS compared to their healthier counterparts. Interestingly, common chronic conditions like obesity, hypertension, or diabetes did not exhibit a comparable association, suggesting a potential psychological influence rather than a solely somatic component. The stressful atmosphere linked to the COVID-19 pandemic emanates not just from the nature of the disease but also from the extensive attention given by mainstream and social media. This dissemination of overwhelming and alarming information is particularly pronounced for populations with comorbidities. ^25– 27^ The persistent perception of threat could potentially trigger HVS in patients recovering from acute COVID-19 pneumonia. This heightened sense of vulnerability may be further exacerbated in individuals with pre-existing respiratory conditions, especially asthma, as supported by the significant association found in our study. ^28, 29^

In our investigation, we failed to identify distinct acute clinical presentations reliably predicting the subsequent onset of HVS. Patients who later developed hyperventilation manifested less severe COVID-19 symptoms and a reduced accumulation of symptom clusters during the acute phase in comparison to those who did not develop HVS. An exception regarding anosmia and dysgeusia should be highlighted. Experiencing these latter symptoms during the acute phase was strongly associated with the occurrence of HVS. Similarly, cognitive impairment also emerged as an independent predictive factor for HVS, as identified in our multivariate regression model with a relative risk RR of 3.47.

These findings prompt considerations regarding a potential organic mechanism for HVS in individuals who have contracted COVID. Multiple studies have highlighted the notable neurotropism of SARS-CoV-2. ^30^ This neural spread rapidly leads to disruptions in taste and olfaction from the onset of infection due to alterations in the neuroperception of gustatory and olfactory chemosensors caused by the virus. ^30– 32^ Moreover, various authors posit that viral neurotropism contributes to the silent hypoxia reported in 32%–65% of infected individuals, affecting both peripheral and central respiratory chemoreceptors. This spread may induce inflammation and impair the processing of hypoxia signals at higher respiratory centers, resulting in inappropriate normal breathing despite severe hypoxia. ^33– 35^ The impairment in the central respiratory command might be proposed as a potential common denominator linking silent hypoxia observed in acute COVID-19 to the later onset of HVS in post-infections. Further neurophysiological studies are imperative to elucidate the precise impact of COVID-19 on chemosensation and nervous respiratory command during and after infection,

Surprisingly, our data do not state any significant association between HVS and markers of COVID-19 infection gravity, including acute COVID-19 form severity, oxygen requirement, radiological extension of pneumonia, and serious complications like ICU admission and pulmonary embolism. Many previous studies also find no substantial correlation between acute pneumonia severity and several clinical and functional outcomes in long COVID-19. ^36, 37^

In the post-infection phase, both patients with and without HVS experience symptom relief and a decrease in the accumulation of symptom clusters, consistent with observations in previous studies on cohorts with long COVID-19. ^1, 37^ Notably, individuals who develop HVS appear to show a slower improvement in symptoms, continuing to experience residual issues such as dyspnea, palpitations, dizziness, and cognitive impairment. These lingering symptoms, often associated with HVS, suggest a significant contribution of HVS to the overall scenario of long COVID-19. ^16, 20, 24^

Of importance, continuous weight loss—a physical sign unrelated to the typical presentation of HVS—predicts the occurrence of HVS by approximately 12-fold in our statistical regression model. This novel finding has not been previously reported in the literature. During the early COVID-19 infection, organic factors such as viral aggression, the hospitalization context, and alterations in taste and smell may affect the energy balance with less food intake and increased energy expenditure, leading to weight loss. ^38, 39^ At the post-infection stage, persistent weight loss would be rather explained by psychological sequelae associated with COVID-19 and the quarantine, which may have increase depression, anxiety, and other mental health issues. ^27, 40, 41^ Restrictive feeding behavior is widely observed in patients with long COVID-19 across all age groups. ^38, 42^ In one prospective study tracking 1230 COVID-19 survivors, Tosato et al. diagnosed malnutrition in 22% of patients 4-5 months after acute disease. Stress can cause changes in appetite, energy, desires, and interests, and at the same time, it is a triggering factor of HVS. ^39, 41^

Furthermore, the PCFS scale may be a valuable tool to suspect HVS in COVID-19 survivors. Our findings identify that individuals displaying functional limitations of grade 3 and 4 are 3.24-fold exposed to developing ventilatory dysfunction during convalescence. Laskovski et al. analyzed PCFS in a longitudinal cohort of 140 COVID-19 survivors and concluded that alterations in PCFS were significantly influenced not only by residual fatigue and dyspnea but also by manifestations of depression and anxiety in COVID-19 survivors, reminding us once again of the HVS manifestations. ^27, 43, 44^

PTSD symptoms among individuals recovering from COVID-19 present an additional explanatory factor for HVS in the post-COVID-19 phase. ^45– 47^ Our multivariate analysis reveals a sixfold increase in the incidence of HVS associated with the presence of PTSD symptoms. A longitudinal observational study in the Netherlands, encompassing 239 patients, highlighted a substantial prevalence of PTSD—37.2% at 3 months and 26.8% at 6 months post-onset of COVID-19 symptoms. ^48^ Similarly, a UK-based online survey, involving 3290 COVID-19 patients, documented a progressive and notable escalation in PTSD prevalence: 15% at 4–8 weeks, 17.3% at 8–12 weeks, and 18.9% at >12 weeks. ^49^

The lasting psychological impact on individuals who have survived the infection may result from the stigma linked with COVID-19, the circumstances of lockdown, and the apprehension of contagion. ^27, 44^ This enduring effect could contribute to prolonged psychological consequences. Ongoing intrusive thoughts or images related to traumatic events experienced during COVID-19, along with flashbacks of hospitalization, including witnessing the demise of other infected individuals, may act as triggers for inappropriate hyperventilation in COVID-19 survivors. ^27, 41, 44^ This rationale aligns with the significant association observed in our study between the PCL-5 score and HVS.

Our study fills a significant gap in the current literature by providing substantial evidence regarding the prevalence of HVS in patients with long COVID. This contributes to a more comprehensive understanding of respiratory dysfunction within this population. We have developed a logistic regression model to assess the likelihood of developing HVS in post-COVID-19 individuals, incorporating predictive factors from both acute and post-infection phases. The prospective design, along with a large sample size, enhances the robustness of our study, minimizing biases and facilitating the generalization of our findings to diverse populations. This could provide valuable assistance to clinicians managing long COVID-19 survivors, aiding in the prompt detection of HVS.

However, a noteworthy limitation is our inability to enhance the accuracy of HVS diagnosis by including a hyperventilation provocation test alongside the Nijmegen questionnaire. ^12^ This limitation stems from the stringent COVID-19 precautions mandated during the pandemic to curb infection spread. Additionally, the study’s follow-up duration was limited to one month for all participants, mainly due to a significant proportion discontinuing their participation or being lost to follow-up after symptom relief.

## Conclusion

HVS manifests in 15.6% of COVID-19 pneumonia survivors, underscoring its significance in the post-acute phase. Recognizing this condition is vital for healthcare providers managing lCOVID-19 cases and follow-up, as it significantly contributes to the array of residual symptoms of post-acute pneumonia. The predictive factors for HVS, identified through our multivariate analysis, highlight the convergence of psychosomatic pathophysiological mechanisms. Further investigations, particularly in neurophysiology and psychology, are imperative to elucidate the precise mechanisms promoting HVS in long COVID-19 patients. The development of individualized management strategies can enhance the care and well-being of individuals grappling with the lasting effects of COVID-19.

## Author contributions

H. Cherif, MR. Charfi conceived the study design. H. cherif carried out patients follow up and data collection. H. Cherif and S. Kalboussi operated the data analysis. H. Cherif, S. Mokaddem, and S. Debbiche drafted the manuscript. F. Yangui and MR. Charfi supervised the research progression. All authors approved the final version of the manuscript.

## Data Availability

Repository name: Zenodo. Title of project: Incidence and Predictive Factors of Hyperventilation Syndrome in Patients after COVID-19 Pneumonia: A Prospective Cohort Study. DOI: 10.5281/zenodo.12668621. ^50^ This project contains the following underlying data:
•
HVS anonym.xlsx Data are available under the terms of the Creative Commons Attribution 4.0 International license (CC-BY 4.0). **Zenodo:** Extended Data, 10.5281/zenodo.14210595. ^51^ This project contains the following underlying data:
• Data file 1: ethic statement HVS • Data file 2: STROBE_checklist_cohort • Data file 3: Data collection form Data are available under the terms of the Creative Commons Attribution 4.0 International license (CC-BY 4.0).
