# Supplementary material for: Incidence and predictive factors of hyperventilation syndrome in patients after COVID 19 pneumonia: a prospective cohort study
Source: F1000Res. 2024 Dec 6;13:1497. [Version 1] doi: 10.12688/f1000research.152196.1 (PMC12000803; doi:10.12688/f1000research.152196.1)
Supplement: Peer review history

# Reviewer report: Reviewer response for version 1

**DOI:** 10.5256/f1000research.166928.r371473

**Published:** 2025-04-15

**Authors:** Nesrine Kallel

**Affiliations:** 1 Faculty of Medicine of Sfax, Sfax, Tunisia

This is a prospective study which aim to determine the incidence and predictive factors of Hyperventilation Syndrome (HVS) in patients after COVID 19 pneumonia. Results share  that the principal factors for developing HVS  are cognitive impairment , persistent weight loss (post-COVID-19 phase), Post-COVID-19 Functional Status (PCFS) grade > 2, and post-traumatic syndrome disorder (PTSD) are independent. The manuscript is clearly presented with different sections. The methods are well explained and the application of statistical analysis is appropriate. The discussion contain a recent published articles. The conclusions align with the topic addressed by the authors.

 This article may be considered for acceptance.

Is the work clearly and accurately presented and does it cite the current literature?

Yes

If applicable, is the statistical analysis and its interpretation appropriate?

Yes

Are all the source data underlying the results available to ensure full reproducibility?

Yes

Is the study design appropriate and is the work technically sound?

Yes

Are the conclusions drawn adequately supported by the results?

Yes

Are sufficient details of methods and analysis provided to allow replication by others?

Yes

Reviewer Expertise:

pulmonologist

I confirm that I have read this submission and believe that I have an appropriate level of expertise to confirm that it is of an acceptable scientific standard.

# Reviewer report: Reviewer response for version 1

**DOI:** 10.5256/f1000research.166928.r371476

**Published:** 2025-03-26

**Authors:** Ahmed Ben saad

**Affiliations:** 1 Faculty of Medicine of Monastir, Monastir, Tunisia

This prospective study aimed to determine the incidence and predictive factors of Hyperventilation Syndrome (HVS) in patients after COVID 19 pneumonia. Authors found that cognitive impairment (acute phase), persistent weight loss (post-COVID-19 phase), Post-COVID-19 Functional Status (PCFS) grade > 2, and post-traumatic syndrome disorder (PTSD) are independent factors for developing HVS. The different sections are clearly presented in the article. The methods are well-explained and the application of statistical analysis is appropriate. Relevant published articles are reported in the discussion. The conclusions align with the topic addressed by the authors. Regarding the  overall incidence of HVS, two values were mentioned: 15.6% and 15.8% ?. Overall, it is a well-presented article. Based on these considerations, it should be considered for acceptance.

Is the work clearly and accurately presented and does it cite the current literature?

Yes

If applicable, is the statistical analysis and its interpretation appropriate?

Yes

Are all the source data underlying the results available to ensure full reproducibility?

Yes

Is the study design appropriate and is the work technically sound?

Yes

Are the conclusions drawn adequately supported by the results?

Yes

Are sufficient details of methods and analysis provided to allow replication by others?

Yes

Reviewer Expertise:

Pulmonology

I confirm that I have read this submission and believe that I have an appropriate level of expertise to confirm that it is of an acceptable scientific standard.

# Response

**Published:** 2025-03-30

**Authors:** Hela CHERIF

**Affiliations:** Pulmonology, University of Tunis El Manar, Tunis, Tunis, Tunisia

**Dear Reviewer,**

 Thank you for your thoughtful and positive feedback on our manuscript. We appreciate your careful review and insightful comments.

 Regarding the incidence of Hyperventilation Syndrome (HVS), the correct value is **15.8%**. The discrepancy was due to a typographical error, which we will correct in the revised version of the manuscript.

 We sincerely appreciate your time and consideration, and we are grateful for your recommendation for acceptance
